# Tobacco Use Status and Temptation to Try E-Cigarettes among a Sample of Appalachian Youth

**DOI:** 10.3390/ijerph18136755

**Published:** 2021-06-23

**Authors:** Delvon T. Mattingly, Jayesh Rai, Osayande Agbonlahor, Kandi L. Walker, Joy L. Hart

**Affiliations:** 1 Department of Communication, University of Louisville, Louisville, KY 40292, USA; delvon@umich.edu (D.T.M.); osayande.agbonlahor@louisville.edu (O.A.); kandi.walker@louisville.edu (K.L.W.); 2 Christina Lee Brown Envirome Institute, School of Medicine, University of Louisville, Louisville, KY 40202, USA; jayesh.rai@louisville.edu; 3 American Heart Association Tobacco Center for Regulatory Science, Dallas, TX 75231, USA

**Keywords:** tobacco, youth, e-cigarettes, Appalachia, temptation

## Abstract

E-cigarettes are commonly used tobacco products among youth populations, including Appalachian youth. However, knowledge of the extent to which tobacco use status relates to temptation to try e-cigarettes is limited. Data from the Youth Appalachian Tobacco Study (*n* = 1047) were used. Temptation to try e-cigarettes was derived from a 12-item situational inventory. Tobacco use status was defined as never, ever non-e-cigarette, and ever e-cigarette use. A factorial ANOVA was used to estimate the adjusted association between tobacco use status and the e-cigarette use temptation scale. Two-way interaction terms between tobacco use status and gender, and tobacco use status and race/ethnicity, were plotted to depict effect modification. Approximately 10% of youth were ever non-e-cigarette users and 24% were ever e-cigarette users. Never and ever non-e-cigarette user middle schoolers had higher temptation to try e-cigarettes than their high school counterparts. The same relationship was found among never and ever e-cigarette users living in households with tobacco users. The ANOVA results suggest a positive, monotonic relationship between tobacco use status and temptation to try e-cigarettes, and that the adjusted group means differ by gender and race/ethnicity. The findings can inform tobacco prevention interventions for youth at higher risk for e-cigarette use, especially youth who have not yet tried e-cigarettes.

## 1. Introduction

Tobacco use is a public health crisis that has warranted attention for decades [1,2]. In recent years, the prevalence of cigarette smoking has decreased among US youth and adults [3,4]. However, this decline has been juxtaposed with an uptick in electronic cigarette (e-cigarette) use, especially among youth [3]. E-cigarettes are devices that deliver nicotine, flavors, and other additives through inhaling vaporized aerosols [1]. The tobacco product landscape is ever evolving, and e-cigarettes today come in many forms, such as traditional disposable devices, tank devices, and pod systems/pod mods [1]. With e-cigarettes and other products, youth are often the target of marketing and advertising by the tobacco industry to cement tobacco use behaviors early and establish the potential for lifetime tobacco purchasing [5]. Thus, it is imperative to better understand the reasons influencing youth decisions to try and continue using these products.

Among US youth, e-cigarettes are the most used tobacco product [6]. In 2020, approximately 13% of US middle and high school students—3.6 million youth—used e-cigarettes in the past 30 days [6]. That same year, the prevalence of cigarette use among youth was 3.3%, slightly lower than the prevalence of cigar use (3.5%) and higher than the prevalence of smokeless tobacco use (2.3%) [6]. Several studies have linked e-cigarette use with subsequent use of combustible tobacco products, with dire implications for deleterious health outcomes; thus, it is important to understand the drivers of use [7,8].

Temptations to use tobacco products may help explain use behaviors. Temptation to smoke among youth refers to the willingness or susceptibility to use tobacco products in particular situations [9]. This construct can be used to identify youth who are susceptible to initiating tobacco product use [9]. Factors, such as the social environment and negative affect, may tempt youth into trying or continuing to use tobacco products [10,11,12]. For example, family and friend smoking is associated with tobacco use among youth, and social context, such as the number of friends who smoke and views on tobacco’s acceptability, is a substantial component to understanding tobacco use behaviors [13,14,15]. In addition, internalizing problems and psychological distress are linked to tobacco initiation and use, with recent empirical evidence establishing a relationship between these factors and e-cigarette use [11,12,16]. Although studies on temptation to use e-cigarettes are limited, the relationship between being tempted to use tobacco and tobacco use shown in other studies is likely similar [17,18,19].

Tobacco use disproportionately burdens certain youth demographic groups. For example, while past-30-day e-cigarette use prevalence in 2020 was similar between male and female youth, the prevalence was substantially higher among White youth (15.5%) than Black youth (6.2%) [6]. In addition, although Black youth have relatively low overall prevalence of tobacco product use, they have the highest prevalence of individual cigar use [6]. Thus, not only is it important to understand determinants of tobacco use, but also how tobacco product use patterns differ by group (e.g., gender, race/ethnicity). Further, tobacco use disparities exist by socioeconomic status and rurality in the United States [20,21]. The 13-state Appalachian region, which ranks high in both poverty and tobacco use, contains disproportionately affected populations of youth who may be differentially exposed to tobacco products [22]. Increased exposure to tobacco products and use may be driven by rural lifestyle, community acceptance of tobacco, and familial involvement in the tobacco trade [22]. Thus, it is not surprising that the prevalence of tobacco product use in the Appalachian region exceeds the national average (20% vs. 16%, respectively) [23]. For example, one study found that smoking remains a significant issue among adults in Central Appalachia, despite substantial declines in smoking rates throughout other US regions during the past decade [24]. These findings also have implications for Appalachian youth as household tobacco use has been linked to tobacco and polytobacco use in this population [25].

The objective of this study is to examine the association between tobacco use status and temptation to try e-cigarettes among a sample of Appalachian youth. The study aims are: (1) to investigate the extent to which tobacco use status is associated with temptation to try e-cigarettes, and (2) to assess the moderating role of gender and race/ethnicity on these associations. One goal is to investigate temptation to try e-cigarettes among youth who have not tried e-cigarettes to shed light on groups potentially at risk for initiation. Another goal is to explore temptation patterns among e-cigarette users to better understand users who may be prone to continued use, including experimenting with additional electronic nicotine delivery systems (ENDS).

## 2. Materials and Methods

### 2.1. Data and Participants

The Youth Appalachian Tobacco Study (YATS) is a cross-sectional analysis of youth residing in the Appalachian regions of Kentucky, North Carolina, and New York from 2014–2016 (*N* = 1280). Appalachian states were selected based on differing youth tobacco use rates. Within each state, we identified Appalachian counties with poverty levels that exceed the national average and their state’s average. From the identified counties, public schools were randomly selected. If a school declined, another was invited to participate. Students in participating schools completed a questionnaire that took approximately 45 min. The questionnaire was distributed during the regular school day and included questions about tobacco use behaviors, tobacco-related perceptions and attitudes, and sociodemographic characteristics. Parental consent and student assent were obtained before data collection. Details on YATS can be found elsewhere [26]. The University of Louisville’s Institutional Review Board approved this research.

Participants who did not respond to the tobacco use variables (*n* = 90), 12 e-cigarette temptation situations (*n* = 81), or relevant covariates, gender (*n* = 16), race/ethnicity (*n* = 36), and household tobacco users (*n* = 10), to be adjusted in the analysis, were excluded. This resulted in an analytic sample of 1047.

### 2.2. Measures

#### 2.2.1. Tobacco Use Status

Tobacco use status was categorized into three groups: never users, ever non-e-cigarette users, and ever e-cigarette users. Participants who reported never using cigarettes, e-cigarettes, or smokeless tobacco were never users; participants who reported ever use of cigarettes or smokeless tobacco but not e-cigarettes were ever non-e-cigarette users; participants who reported ever use of e-cigarettes were ever e-cigarette users. For simplicity, we refer to ever non-e-cigarette and ever e-cigarette users as non-e-cigarette and e-cigarette users. These categories were selected to evaluate the unique experiences of youth who have never used tobacco, youth who have used tobacco but not e-cigarettes, and youth who have used tobacco including e-cigarettes, and how these experiences relate to temptation to try e-cigarettes.

#### 2.2.2. E-Cigarette Use Temptation Scale

Youth who participated in YATS indicated whether they were tempted to try e-cigarettes in the following twelve situations: (1) while talking and relaxing; (2) when things are not going my way and I am frustrated; (3) with friends at a party; (4) when others are talking about how much they like e-cigarettes; (5) when I am afraid I might gain weight; (6) while having a good time; (7) when I am very anxious and stressed; (8) when I want to fit in with a crowd; (9) when I want to know how an e-cigarette tastes; (10) when I want to lose weight; (11) when my friends ask if I want to try an e-cigarette; (12) when it is difficult to refuse an e-cigarette. The first ten situations were derived from the validated Situational Temptations Inventory for Smoking, which is highly reliable [9,27]. The situations in this inventory contain measures on positive social and negative affect situations, habit strength, and weight control [9]. Although the inventory is often used in smoking cessation programs to prevent smoking relapse [9,19], it can be used to identify patterns of initiation and continued use among younger populations. YATS included situations 11 and 12 to add social situations relevant to peer influence and tobacco use. For the twelve situations, response options ranged from 1) “not tempted at all” to 5) “extremely tempted.” An e-cigarette use temptation scale was created by summing the twelve situation scores (range: 12–60), with a score of 12 indicating youth who responded “not tempted at all” to each situation, and a score of 60 indicating youth who responded “extremely tempted” to each situation. Distributions and means of each e-cigarette temptation situation and the e-cigarette use temptation scale are presented in Supplementary Table S1. A Cronbach’s alpha was calculated to test the internal reliability of the twelve situations among the entire sample, with a value of 0.96 indicating excellent reliability (see Supplementary Table S2).

#### 2.2.3. Covariates

The following demographic characteristics were included in this analysis: age, gender (male or female), race/ethnicity (White or racial/ethnic minority groups), school type (middle or high school), state (Kentucky, North Carolina, or New York), and number of household tobacco users (zero or one or more). The racial/ethnic minority group included self-reported Hispanic, African American, Asian, and other participants.

#### 2.2.4. Statistical Analysis

The distributions of sociodemographic characteristics overall, and by tobacco use status, were computed. Chi-square and student’s t-tests evaluated three pairwise differences between: (1) never users and e-cigarette users; (2) never users and non-e-cigarette users; (3) non-e-cigarette users and e-cigarette users. Frequencies and means of the e-cigarette use temptation scale were calculated by each participant characteristic overall and by tobacco use status. Within-group differences were estimated using Wilcoxon rank-sum tests for binary sociodemographic characteristics and Kruskal–Wallis tests for nominal sociodemographic characteristics overall and within each tobacco use group. To estimate the effect of tobacco use status on temptation to try e-cigarettes, a factorial ANOVA was used, adjusting for gender, race/ethnicity, school type, state, and household tobacco users. This model included five two-way interaction terms between tobacco use status and each covariate (Supplementary Table S3). Each interaction term was evaluated for its statistical significance using Wald tests and alpha levels of 0.05. Factors that approached or were statistically significant were retained in a parsimonious factorial ANOVA, excluding each two-way interaction between tobacco use status and school type, state, and household tobacco users, respectively. For each remaining interaction term, average marginal effects (adjusted group means) and associated 95% confidence intervals were plotted to display the relationship between tobacco use status and temptation to try e-cigarettes by gender and race/ethnicity, respectively, using the margins command in Stata. Data were analyzed using Stata 16.1 (StataCorp, College Station, TX, USA).

## 3. Results

### 3.1. Sociodemographic Characteristics by Tobacco Use Status

Table 1 presents the distribution of sociodemographic characteristics by tobacco use status. Of the sample, approximately half were male (51.1%), the majority were White (88.6%), the mean age was 13.8 (SD: 1.9), over half were in middle school (59.9%), the majority lived in North Carolina (45.5%) or Kentucky (34.7%), and over half (57.1%) had at least one household tobacco user. Approximately two-thirds (65.4%) were never users, and 10.5% and 24.1% were non-e-cigarette and e-cigarette users, respectively. Tobacco use status differed by all sociodemographic characteristics except race/ethnicity. Notably, never users and e-cigarette users significantly differed in age (*p* < 0.001), school type (*p* < 0.001), state (*p* < 0.001), and household tobacco users (*p* < 0.001). Similar associations were found comparing never users and non-e-cigarette users, with an additional statistically significant finding by gender (*p* = 0.017). Non-e-cigarette users and e-cigarette users differed only by state (*p* < 0.001).

### 3.2. Temptation to Try E-Cigarettes by Sociodemographic Characteristics 

The overall mean of the e-cigarette use temptation scale was 19.27 (95% CI: 18.57–19.98) (see Table 2). Means significantly differed by school type (*p* = 0.002), state (*p* < 0.001), household tobacco users (*p* < 0.001), and tobacco use status (*p* < 0.001). 

Distributions of the e-cigarette use temptation scale within each tobacco use group by sociodemographic characteristics are presented in Table 3. Among never users, means differed between youth with zero household members who use tobacco (mean: 14.18, 95% CI: 13.56–14.81) and youth with one or more household members who use tobacco (mean: 15.56, 95% CI: 14.71–16.41) (*p* = 0.048). Among non-e-cigarette users, means differed by school type, where middle schoolers had a higher temptation to try e-cigarettes (mean: 22.15, 95% CI: 18.71–25.59) than high schoolers (mean: 16.74, 95% CI: 14.65–18.82) (*p* = 0.007). Among e-cigarette users, means differed by household tobacco users, where youth with zero household members who use tobacco (mean: 27.96, 95% CI: 25.05–30.88) reported less temptation to try e-cigarettes than youth with one or more household members who use tobacco (mean: 32.63, 95% CI: 30.60–34.66) (*p* = 0.024)

### 3.3. Factorial ANOVA of Main and Interaction Effects

Results from the factorial ANOVA (see Table 4) show that the adjusted group means for temptation to try e-cigarettes statistically differ by tobacco use status (*p* < 0.001), state (*p* = 0.017), and household tobacco users (*p* = 0.007). 

**Figure 1:**
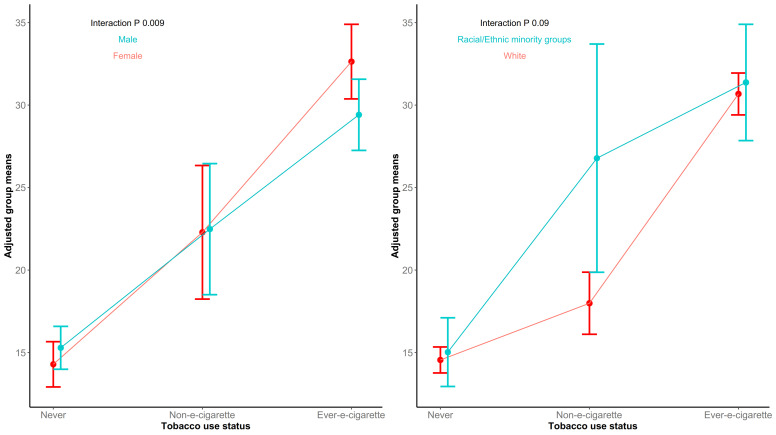
Average Marginal Effects of Two-Way Interactions between Tobacco Use Status and Gender and Between Tobacco Use Status and Race/Ethnicity.

Figure 1 displays the interaction between tobacco use status and gender (left), and the interaction between tobacco use status and race/ethnicity (right), on temptation to try e-cigarettes. Though the two-way interaction term between tobacco use status and race/ethnicity was attenuated after fitting a parsimonious factorial ANOVA, adjusted group means suggest that White and racial/ethnic minority never users and e-cigarette users have a similar temptation to try e-cigarettes. However, among non-e-cigarette users, racial/ethnic minority youth had higher adjusted group means for temptation to try e-cigarettes (mean: 26.78, 95% CI: 19.87, 33.70) than White youth (mean: 17.99, 95% CI: 16.11, 19.87). Adjusted group means for temptation to try e-cigarettes were plotted for males and females at each level of tobacco use status. Results indicate that male never users had similar adjusted group means for temptation to try e-cigarettes (mean: 15.29, 95% CI: 13.99, 16.59) to female never users (mean: 14.29, 95% CI: 12.92, 15.66). Similar results were observed for male and female non-e-cigarette users. In contrast, among e-cigarette users, females have higher adjusted group means for temptation to try e-cigarettes (mean: 32.64, 95% CI: 30.38–34.90) than males (mean: 29.41, 95% CI: 27.26–31.57). Adjusted group means and associated 95% CIs are presented in Supplementary Table S4.

## 4. Discussion

To our knowledge, this study is the first to examine the relationship between tobacco use status and temptation to try e-cigarettes among youth. We found substantial within-group variation; for example, among non-e-cigarette users, temptation to try e-cigarettes was higher in middle schoolers than high schoolers, possibly indicating that youth tobacco users may be swayed at younger ages. This finding might also suggest that older non-e-cigarette users may have entrenched tobacco use behavior and are less likely to try additional products. However, temptation to try e-cigarettes was generally lower in middle schoolers than high schoolers, suggesting that the aforementioned relationship may only apply to middle schoolers who use cigarettes or smokeless tobacco. In addition, previous research indicates that e-cigarette users are at risk for subsequent combustible tobacco use and that combustible tobacco users are at risk for subsequent e-cigarette use, raising the potential for dual use in both groups [28]. Thus, tobacco control efforts should remain vigilant in addressing potential initiation of e-cigarette use among youth of all ages. 

Youth never users and e-cigarette users with household members who use tobacco reported greater temptation to try e-cigarettes, which corroborates existing literature suggesting that familial tobacco use or endorsement is directly associated with use among youth in the same household [13,29,30]. We did not observe a difference among youth non-e-cigarette users, possibly highlighting an important variation based on tobacco products used by parents or siblings in the household. For example, youth who use cigarettes or smokeless tobacco may be less tempted to use e-cigarettes because others in their household are not using them.

The statistical interaction between tobacco use status and race/ethnicity suggests that non-e-cigarette users from racial/ethnic minorities may have higher susceptibility to being tempted to use e-cigarettes. Although the racial/ethnic minority category is heterogeneous, these findings indicate that Appalachian youth from racial/ethnic minorities who use cigarettes or smokeless tobacco may be at higher risk for trying e-cigarettes than their White counterparts, putting them at elevated risk for additional nicotine consumption and possible health consequences [31,32]. The statistical interaction between tobacco use status and gender shows that female never users have lower temptation to try e-cigarettes than male never users, yet female e-cigarette users have higher temptation to try e-cigarettes than male e-cigarette users. Thus, among these youth, temptations vary by use status and gender; however, variation in tobacco product use by sociodemographic groups (e.g., gender, race/ethnicity) warrants further investigation to better understand potential influences on initiation or continued use [3,6,25], and our results emphasize the complexity by which these relationships occur.

We acknowledge the limitations associated with the study. First, responses were self-reported and subject to potential associated biases. Second, 18.2% of participants had missing data on the exposure, outcome, or covariates, which may have influenced the results. In addition, the sample size of minority youth non-e-cigarette users may have precluded detecting meaningful interactions due to a lack of statistical power. Third, the findings may not be generalizable across Appalachia given that our study focused on three states. Fourth, our findings may be more clear-cut for never and non-e-cigarette users than e-cigarettes users, as the latter group had tried e-cigarettes. That is, temptation to try e-cigarettes among never and non-e-cigarette users may identify subsets of users who are more at risk for trying these products. However, we assume that e-cigarette users who are tempted to use e-cigarettes are more susceptible to continued use or trying various ENDS, but additional research is needed to better understand the mechanisms behind temptation to continue or expand use. Moreover, during the data collection period, changes in perceptions or use behavior may have occurred and not be fully reflected in the findings. Additionally, study findings do not reflect changes since our data collection, such as shifts potentially influenced by the COVID-19 pandemic. Nevertheless, this study is among the first to examine the relationship between tobacco use status and temptation to try e-cigarettes in a sample of Appalachian youth.

## 5. Conclusions

Substantial variation in the extent to which tobacco use status is associated with temptation to try e-cigarettes exists overall and by sociodemographic groups, such as gender and race/ethnicity. Importantly, our results suggest that non-e-cigarette users from racial/ethnic minority groups may find themselves in situations that tempt them to try e-cigarettes more than their White counterparts. Tailored tobacco prevention efforts for groups at-risk of initiating or continuing e-cigarette use, such as Appalachian youth, are especially important. Refinements to such efforts based on sociodemographics may contribute to overall effectiveness, thus decreasing the likelihood of negative long-term health consequences that come with e-cigarette use.

## Figures and Tables

**Table 1:** Participant Characteristics by Tobacco Use Status, The Youth Appalachian Tobacco Study (*n* = 1047).

		Tobacco Use Status, *n* (%)			
	Total	Never	Ever Non-E-Cigarette	Ever E-Cigarette	
Characteristics	1047 (100.0)	685 (65.4)	110 (10.5)	252 (24.1)	*p*-value
Gender					0.18 ^a^
Male	535 (51.1)	333 (48.6)	67 (60.9)	135 (53.6)	0.017 ^b^
Female	512 (48.9)	352 (51.4)	43 (39.1)	117 (46.4)	0.20 ^c^
Race/ethnicity					0.47 ^a^
White	928 (88.6)	600 (87.6)	103 (93.6)	225 (89.3)	0.07 ^b^
Racial/ethnic minority groups	119 (11.4)	85 (12.4)	7 (6.4)	27 (10.7)	0.19 ^c^
Age					<0.001 ^a^
Mean ± SD	13.8 ± 1.9	13.3 ± 1.8	14.7 ± 2.0	14.6 ± 1.8	<0.001 ^b^
Median (min–max)	14 (11–19)	13 (11–19)	14 (11–19)	15 (11–19)	0.75 ^c^
School type					<0.001 ^a^
Middle school	627 (59.9)	468 (68.3)	53 (48.2)	106 (42.1)	<0.001 ^b^
High school	420 (40.1)	217 (31.7)	57 (51.8)	146 (57.9)	0.28 ^c^
State					0.002 ^a^
New York	208 (19.9)	200 (29.2)	20 (18.2)	36 (14.3)	<0.001 ^b^
Kentucky	363 (34.7)	333 (48.6)	62 (56.4)	101 (40.1)	0.001 ^c^
North Carolina	476 (45.5)	152 (22.2)	28 (25.4)	115 (45.6)	
Household tobacco users (count) ^d^					<0.001 ^a^
Zero	449 (42.9)	338 (49.3)	32 (29.1)	79 (31.4)	<0.001 ^b^
1+	598 (57.1)	347 (50.7)	78 (70.9)	173 (68.6)	0.67 ^c^

^a^ Chi-square test comparing never users to ever e-cigarette users. ^b^ Chi-square test comparing never users to ever non-e-cigarette users. ^c^ Chi-square test comparing ever non-e-cigarette users to ever e-cigarette users. ^d^ Number does not include the participants.

**Table 2:** Distribution of the E-cigarette Use Temptation Scale by Sociodemographic Characteristics.

Characteristics	*n*	Mean (95% CI)	*p* ^a^
Overall	1047	19.27 (18.57, 19.98)	
Gender			0.55
Male	535	19.45 (18.49, 20.42)	
Female	512	19.08 (18.04, 20.11)	
Race/ethnicity			0.84
White	928	19.25 (18.51, 20.00)	
Racial/ethnic minority groups	119	19.39 (17.22, 21.57)	
School type			0.002
Middle school	627	18.53 (17.64, 19.43)	
High school	420	20.37 (19.22, 21.51)	
State			<0.001
New York	208	16.57 (15.27, 17.88)	
Kentucky	363	21.32 (19.99, 22.65)	
North Carolina	476	18.88 (17.88, 19.89)	
Household tobacco users (count)			<0.001
Zero	449	16.95 (16.08, 17.82)	
1+	598	21.01 (19.99, 22.03)	
Tobacco use status			<0.001
Never	685	14.88 (14.35, 15.41)	
Ever non-e-cigarette use	110	19.35 (17.33, 21.36)	
Ever e-cigarette use	252	31.17 (29.49, 32.84)	

^a^ Chi-square test comparing sociodemographic characteristics by the e-cigarette use temptation scale.

**Table 3:** Distribution of the E-cigarette Use Temptation Scale Within Each Tobacco Use Group by Sociodemographic Characteristics.

	Tobacco Use Status								
	Never			Ever Non-E-Cigarette			Ever E-Cigarette		
Characteristics	*n*	Mean (95% CI)	*p* ^a^	*n*	Mean (95% CI)	*p* ^a^	*n*	Mean (95% CI)	*p* ^a^
Overall	685	14.88 (14.35, 15.41)		110	19.35 (17.33, 21.36)		252	31.17 (29.49, 32.84)	
Gender			0.99			0.93			0.12
Male	333	15.37 (14.52, 16.23)		67	19.27 (16.66, 21.88)		135	29.61 (27.50, 31.72)	
Female	352	14.42 (13.77, 15.06)		43	19.47 (16.18, 22.75)		117	32.96 (30.29, 35.62)	
Race/ethnicity			0.54			0.22			0.89
White	600	14.88 (14.31, 15.45)		103	18.76 (16.83, 20.68)		225	31.14 (29.36, 32.92)	
Racial/ethnic minority groups	85	14.88 (13.33, 16.44)		7	6.69 (11.63, 44.37)		27	31.37 (26.14, 36.60)	
School type			0.77			0.007			0.13
Middle school	468	14.98 (14.32, 15.63)		53	22.15 (18.71, 25.59)		106	32.43 (29.68, 35.19)	
High school	217	14.68 (13.77, 15.58)		57	16.74 (14.65, 18.82)		146	30.25 (28.14, 32.35)	
State			0.16			0.13			0.07
New York	152	14.30 (13.17, 15.42)		20	14.90 (12.03, 17.77)		36	27.11 (22.71, 31.51)	
Kentucky	200	15.45 (14.41, 16.49)		28	19.79 (15.28, 24.29)		101	33.41 (30.61, 36.20)	
North Carolina	333	14.81 (14.07, 15.55)		62	20.58 (17.76, 23.40)		115	30.47 (28.10, 32.84)	
Household tobacco users (count)			0.048			0.73			0.024
Zero	338	14.18 (13.56, 14.81)		32	18.97 (15.64, 22.30)		79	27.96 (25.05, 30.88)	
1+	347	15.56 (14.71, 16.41)		78	19.50 (16.97, 22.03)		173	32.63 (30.60, 34.66)	

^a^ Chi-square test comparing sociodemographic characteristics within each tobacco use status.

**Table 4:** Factorial ANOVA of Main and Interaction Effects of Sociodemographic Characteristics and Tobacco Use Status on Temptation to Use E-cigarettes.

Source of Variance	Partial Sum of Squares	df	Mean Square	F	*p*
Tobacco use status	18,712.71	2	9356.35	108.65	<0.001
Gender	68.01	1	68.01	0.79	0.37
Race/ethnicity	471.31	1	471.31	5.47	0.020
School type	245.84	1	245.84	2.85	0.09
State	709.28	2	354.64	4.12	0.017
Household tobacco users	637.10	1	637.10	7.40	0.007
Tobacco use status * gender	817.34	2	408.67	4.75	0.009
Tobacco use status * race/ethnicity	412.10	2	206.05	2.39	0.09
Residual	89,043.50	1034	86.12		

* Refers to interaction term.

## Data Availability

The data used in this study are available on request from the corresponding author.

## Supplementary Materials

The following are available online at https://www.mdpi.com/article/10.3390/ijerph18136755/s1, Supplementary Table S1: Distributions and Means of Each E-cigarette Use Temptation Situation and the E-cigarette Use Temptation Scale (*n* = 1047); Supplementary Table S2: Test for Internal Reliability of the 12 E-cigarette Use Temptation Items; Supplementary Table S3: Factorial ANOVA of Main and Interaction Effects of Sociodemographic Characteristics and Tobacco Use Status on Temptation to Use E-cigarettes; Supplementary Table S4: Adjusted Group Means of the Relationship Between Tobacco Use Status and Temptation to Try E-cigarettes by Level of Gender or Race/Ethnicity.
