# Supplementary material for: Tobacco Use Status and Temptation to Try E-Cigarettes among a Sample of Appalachian Youth
Source: Int J Environ Res Public Health. 2021 Jun 23;18(13):6755. doi: 10.3390/ijerph18136755 (PMC8267625; doi:10.3390/ijerph18136755)
Supplement: Supplementary file 1 [file ijerph-18-06755-s001.zip › ijerph-1215949-supplementary.pdf]

Supplementary Table S1. Distributions and Means of Each E-cigarette Use Temptation Situation and the E-cigarette Use Temptation Scale (n=1047)

	E-cigarette Use Temptations Response Options, n (%)					
	Not tempted at all	2	3	4	Extremely tempted	Mean (95% CI)
**I am tempted to try electronic cigarettes:**						
1. While talking and relaxing	800 (76.4)	65 (6.2)	92 (8.8)	46 (4.4)	44 (4.2)	1.53 (1.47, 1.60)
2. When things are not going my way and I am frustrated	737 (70.4)	86 (8.2)	106 (10.1)	56 (5.4)	62 (5.9)	1.68 (1.61, 1.76)
3. With friends at a party	739 (70.6)	69 (6.6)	110 (10.5)	61 (5.8)	68 (6.5)	1.71 (1.64, 1.79)
4. When others are talking about how much they like e-cigarettes	771 (73.6)	76 (7.3)	102 (9.7)	53 (5.1)	45 (4.3)	1.59 (1.52, 1.66)
5. When I am afraid I might gain weight	837 (79.9)	71 (6.8)	84 (8.0)	23 (2.2)	32 (3.1)	1.42 (1.36, 1.47)
6. While having a good time	747 (71.4)	60 (5.7)	109 (10.4)	61 (5.8)	70 (6.7)	1.71 (1.63, 1.78)
7. When I am very anxious and stressed	739 (70.6)	70 (6.7)	111 (10.6)	56 (5.4)	71 (6.8)	1.71 (1.64, 1.79)
8. When I want to fit in with a crowd	798 (76.2)	89 (8.5)	96 (9.2)	33 (3.2)	31 (3.0)	1.48 (1.42, 1.54)
9. When I want to know how an e-cigarette tastes	715 (68.3)	74 (7.1)	103 (9.8)	89 (8.5)	66 (6.3)	1.77 (1.70, 1.85)
10. When I want to lose weight	844 (80.6)	66 (6.3)	86 (8.2)	18 (1.7)	33 (3.2)	1.40 (1.35, 1.46)
11. When my friends ask if I want to try an e-cigarette	748 (71.4)	69 (6.6)	119 (11.4)	53 (5.1)	58 (5.5)	1.67 (1.59, 1.73)
12. When it is difficult to refuse an e-cigarette	785 (75.0)	63 (6.0)	106 (10.1)	33 (3.2)	60 (5.7)	1.59 (1.52, 1.66)
**E-cigarette Use Temptation Scale**						19.27 (18.6, 20.0)

Supplementary Table S2. Test for Internal Reliability of the 12 E-cigarette Use Temptation Items

	Cronbach's alpha
**I am tempted to try electronic cigarettes…**	
1. While talking and relaxing	0.9607
2. When things are not going my way and I am frustrated	0.9596
3. With friends at a party	0.9590
4. When others are talking about how much they like e-cigarettes	0.9586
5. When I am afraid I might gain weight	0.9627
6. While having a good time	0.9594
7. When I am very anxious and stressed	0.9592
8. When I want to fit in with a crowd	0.9612
9. When I want to know how an e-cigarette tastes	0.9605
10. When I want to lose weight	0.9626
11. When my friends ask if I want to try an e-cigarette	0.9586
12. When it is difficult to refuse an e-cigarette	0.9597
**E-cigarette Use Temptation Scale**	**0.9634**

Supplementary Table S3. Factorial ANOVA of Main and Interaction Effects of Sociodemographic Characteristics and Tobacco Use Status on Temptation to Use E-cigarettes

Source of Variance	Partial Sum of Squares	df	Mean Square	F	*P*
Tobacco use status	15131.60	2	7565.80	88.62	<0.001
Gender	64.41	1	64.41	0.75	0.38
Race/ethnicity	587.70	1	587.70	6.88	0.009
School type	460.99	1	460.99	5.40	0.020
State	896.07	2	448.04	5.25	0.005
Household tobacco users	218.40	1	218.40	2.56	0.11
Tobacco use status*gender	795.55	2	397.78	4.66	0.010
Tobacco use status*race/ethnicity	507.56	2	253.78	2.97	0.052
Tobacco use status*school type	355.29	2	177.64	2.08	0.13
Tobacco use status*state	545.30	4	136.32	1.60	0.17
Tobacco use status*household tobacco users	388.00	2	194.00	2.27	0.10
Residual	87594.89	1,026	85.38		

[*] refers to interaction term

Supplementary Table S4. Adjusted Group Means of the Relationship Between Tobacco Use Status and Temptation to Try E-cigarettes by Level of Gender or Race/Ethnicity

Interactions	Adjusted Group Means	95% CI	*P*
**Tobacco Use Status and Gender**			
Never use*female	14.29	12.92, 15.66	<0.001
Never use*male	15.29	13.99, 16.59	<0.001
Ever non-e-cigarette use*female	22.29	18.24, 26.34	<0.001
Ever non-e-cigarette use*male	22.48	18.51, 26.46	<0.001
Ever e-cigarette use*female	32.64	30.38, 34.90	<0.001
Ever e-cigarette use*male	29.41	27.26, 31.57	<0.001
**Tobacco Use Status and Race/Ethnicity**			
Never use*white	14.55	13.76, 15.34	<0.001
Never use*racial/ethnic minority groups	15.03	12.94, 17.11	<0.001
Ever non-e-cigarette use*white	17.99	16.11, 19.87	<0.001
Ever non-e-cigarette use*racial/ethnic minority groups	26.78	19.87, 33.70	<0.001
Ever e-cigarette use*white	30.68	29.41, 31.95	<0.001
Ever e-cigarette use*racial/ethnic minority groups	31.37	27.85, 34.90	<0.001

* refers to interaction term
